# CRISPR knockout rat cytochrome P450 3A1/2 model for advancing drug metabolism and pharmacokinetics research

**DOI:** 10.1038/srep42922

**Published:** 2017-02-20

**Authors:** Jian Lu, Yanjiao Shao, Xuan Qin, Daozhi Liu, Ang Chen, Dali Li, Mingyao Liu, Xin Wang

**Affiliations:** 1 Shanghai Key Laboratory of Regulatory Biology, Institute of Biomedical Sciences and School of Life Sciences, East China Normal University, Shanghai, China; 2 Center for Translational Cancer Research, Institute of Biosciences and Technology, Texas A&M University Health Science Center, Houston, Texas, USA

## Abstract

Cytochrome P450 (CYP) 3A accounts for nearly 30% of the total CYP enzymes in the human liver and participates in the metabolism of over 50% of clinical drugs. Moreover, CYP3A plays an important role in chemical metabolism, toxicity, and carcinogenicity. New animal models are needed to investigate CYP3A functions, especially for drug metabolism. In this report, *Cyp3a1/2* double knockout (KO) rats were generated by CRISPR-Cas9 technology, and then were characterized for viability and physiological status. The *Cyp3a1/2* double KO rats were viable and fertile, and had no obvious physiological abnormities. Compared with the wild-type (WT) rat, *Cyp3a1/2* expression was completely absent in the liver of the KO rat. *In vitro* and *in vivo* metabolic studies of the CYP3A1/2 substrates indicated that CYP3A1/2 was functionally inactive in double KO rats. The *Cyp3a1/2* double KO rat model was successfully generated and characterized. The *Cyp3a1/2* KO rats are a novel rodent animal model that will be a powerful tool for the study of the physiological and pharmacological roles of CYP3A, especially in drug and chemical metabolism *in vivo*.

The Cytochrome P450 (CYP) enzymes play an essential role in the biotransformation of endogenous molecules and xenobiotics^1^. Today, almost 75% of drugs on the market are bio-transformed through CYP-mediated metabolism and the main CYP isoforms, CYP3A4/5, CYP2C9/19, CYP2D6, CYP1A1/2 and CYP2E1, participate in nearly 95% of the reactions^2^. In particular, CYP3A4, as the most abundant CYP isoform in the human liver and intestine, is involved in the phase I transformation of toxins, carcinogens, bile acids, steroid hormones, and more than 50% of the drugs used in the clinic^3^,^4^. Moreover, CYP3A4 is either induced or inhibited by many chemicals, which influences the elimination of co-administrated drugs, leading to therapy failure or unwanted toxicity^4^,^5^.

A variety of single CYP enzyme-null mouse models, including Cyp3a, have been reported as potent and precise tools used to illustrate the potential functions of CYP isoforms on the metabolism, toxicity and carcinogenicity of chemicals^6^,^7^. Until now, however, *Cyp3a* gene knockout (KO) rats have not been reported due to the complexity and limitation of gene editing techniques. Compared with the *Cyp3a* KO mouse model, *Cyp3a* KO rat model is more important to pharmacological research, especially drug metabolism and pharmacokinetic (DMPK) studies. On the one hand, the rat is larger in size, and possesses more blood, compared with the mouse. Moreover, rats in some disease models such as breast cancer are physiologically more similar to humans than mice^8^,^9^. Therefore, the *Cyp* KO rat model could be a good supplement to the *Cyp* KO mouse model, overcoming some disadvantages of the mouse model. On the other hand, since many CYP isoforms expressed in different species possess different substrate affinities, it is very difficult to extrapolate the results from one specific animal species to humans^4^. Therefore, results from multiple animals should be taken into consideration.

Recently, application of the Clustered Regularly Interspaced Short Palindromic Repeats (CRISPR)/CRISPR-associated 9 (CRISPR-Cas9) system from *Streptococcus pyogenes* has greatly reduced the difficulties of genome editing in various species including the rat^10^,^11^,^12^. The CRISPR-Cas9 system consists of a non-specific nuclease, Cas9 protein, and a single guide RNA (sgRNA) that directs Cas9 protein to the target sites using the rules of Watson-Crick base-pairing^8^,^11^. Compared with previous techniques, the CRISPR-Cas9 system shows distinct advantages in editing multiple genes simultaneously^10^,^13^. To take advantages of rats in DMPK and disease research and to enrich resources of animal model in pharmacology, we want to generate a *Cyp3a1* and *3a2* double KO rat model via the CRISPR-Cas9 system.

In this study, we successfully created a *Cyp3a1/2* double KO rat model using the CRISPR-Cas9 system. *Cyp3a1/2* double KO rats were characterized for viability and physiological status. The absence of *Cyp3a1/2* expression in rat liver and intestine was confirmed by both PCR analysis of hepatic cDNA and immunohistochemical analysis. Further *in vitro* and *in vivo* metabolic studies of the CYP3A1/2 substrates were conducted to verify that CYP3A1/2 was functionally inactive in KO rats. The *Cyp3a1/2* double KO rat was viable, fertile, physiological normal and presented impaired metabolic ability towards selected CYP3A probe substrates.

## Results

### Generation of *Cyp3a1* and *Cyp3a2* double KO rats using CRISPR-Cas9

**Figure 1:**
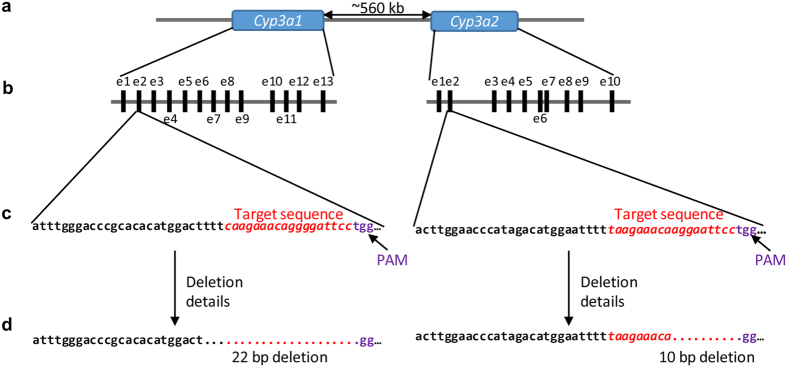
The strategy for generation of the *Cyp3a1/2* double knockout rat model. (**a**) Schematic representation of the chromosomal organization of *Cyp3a1* and *Cyp3a2*. (**b**) Exon/intron structure of *Cyp3a1* and *Cyp3a2*. Exons are represented as 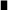, ‘e’, indicates ‘exon’. (**c**) Targets selected for the deletion of *Cyp3a1* and *Cyp3a2*. (**d**) Resulting deletions in the animal model.

**Figure 2:**
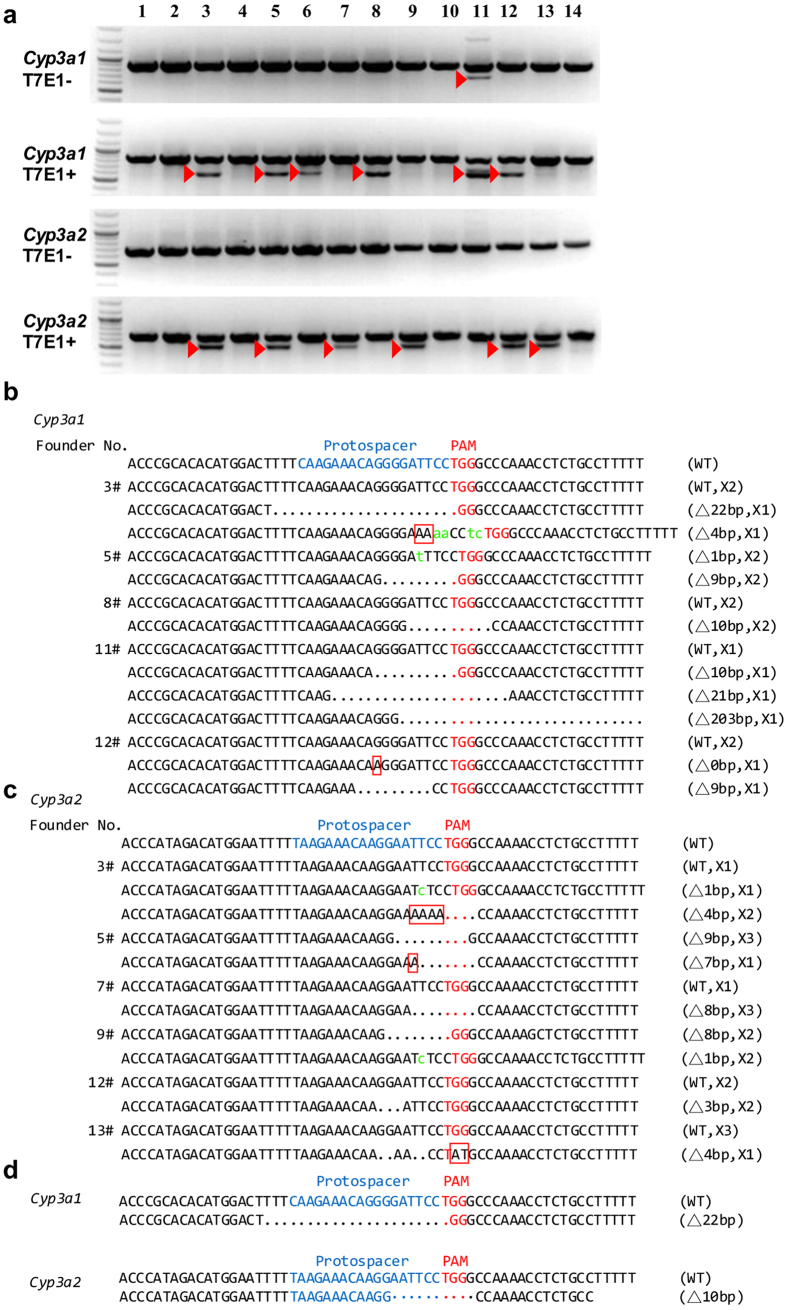
Genotyping of *Cyp3a1/2* double KO rats generated by the CRISPR-Cas9 system. (**a**) Detection of the mutations in the F_0_ generation for *Cyp3a1/2* by T7E I digestion using PCR products amplified from F_0_ rats tail genomic DNA by Primer No. 1 and 2 (Table 3). T7E I−, before T7E I digestion. T7E I+, after T7E I digestion. 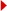, mutant band. DNA sequencing of (**b**) *Cyp3a1* or (**c**) *Cyp3a2* genomic loci in F_0_ rats. Four TA clones of the PCR products amplified from each F_0_ rat were sequenced. “. ”, nucleotide deletion. Lowercase letter, nucleotide insertion. 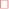, Red box, nucleotide substitution. “▵”, the number of changed nucleotide. “X”, the number of each genotype in four clones. (**d**) The details of mutations in F_2_ generation.

To investigate the role of *Cyp3a* in drug metabolism, we generated rats with CRISPR-Cas9-mediated disruption in both isoforms of this gene. For targeting *Cyp3a1,* we selected 5′-CAAGAAACAGGGGATTCC-3′ followed by TGG as the target site, and 5′-TAAGAAACAAGGAATTCC-3′ followed by TGG for targeting *Cyp3a2*. The targeting strategy is shown in Fig. 1. A mixture of *Cyp3a1* sgRNA (25 ng/μL), *Cyp3a2* sgRNA (25 ng/μL) and Cas9 mRNA (50 ng/μL) was co-microinjected into one-cell fertilized eggs of Sprague-Dawley (SD) rats and 14 progenies were born. To identify the gene modifications of the F_0_ generation, the targeted loci of *Cyp3a1* and *Cyp3a2* were PCR amplified and T7E I (T7 endonuclease I) cleavages were detected in rat #3, #5, #6 (unexpected death at day 11), #8, #11 and #12 founders for *Cyp3a1* and in #3, #5, #7, #9, #12 and #13 founders for *Cyp3a2* (Fig. 2a), which indicated the potential for genome modification at targeted loci. We sequenced these regions and confirmed these modifications (Fig. 2b,c). For *Cyp3a1*, founders #3, #5, #8 and #11 contained at least one frame-shifting mutation; frame-shift mutations were also observed for *Cyp3a2* in founders #3, #5, #7, #9 and #13. Thus, founders #3 and #5 were crossed with wild-type (WT) rats and the genotypes of F_1_ offspring were determined. For *Cyp3a1*, several F_1_ progeny generated from F_0_-#3, five progeny out of eleven total pups (#3, #4, #7, #8 and #11) showed the same nucleotide deletion (deletion of 22 bp) as F_0_-#3 (Fig. 2d). Nevertheless, the genotype with nucleotide insertion and substitution was not transmitted from F_0_-#3 which may be due to genetic mosaicism caused by the CRISPR/Cas9 system^10^,^14^. For *Cyp3a2*, in the F_1_ generated from F_0_-#3, again five pups out of eleven (#1, #3, #4, #8 and #11) possessed a 10 bp nucleotide deletion (Fig. 2d), which was not even detected in F_0_-#3. This inconsistency suggested that sequencing 4 clones of PCR products from F_0_ tail DNA may be not enough to reveal all genetic modifications^14^. In conclusion, progeny #3, #4, #8 and #11 were identified as *Cyp3a1* and *Cyp3a2* double KO F_1_ rats by directly sequencing of the PCR products from rat tail DNA. However, in the F_1_ generation of F_0_-5#, no *Cyp3a1* and *Cyp3a2* double KO F_1_ rat was obtained (Data not shown). To generate *Cyp3a1* and *Cyp3a2* double KO and homozygous rats, F_1_-3#(♀) was mated with F_1_-4#(♂), and F_1_-8#(♀) was crossed with F_1_-11#(♂) to generate the F_2_ generation. The genomic modification in F_1_ was transmitted to the F2 generation efficiently and stably with the described mutations in *Cyp3a1* and *Cyp3a2* gene (Fig. 2d).

### Off-target analysis

**Figure 3:**
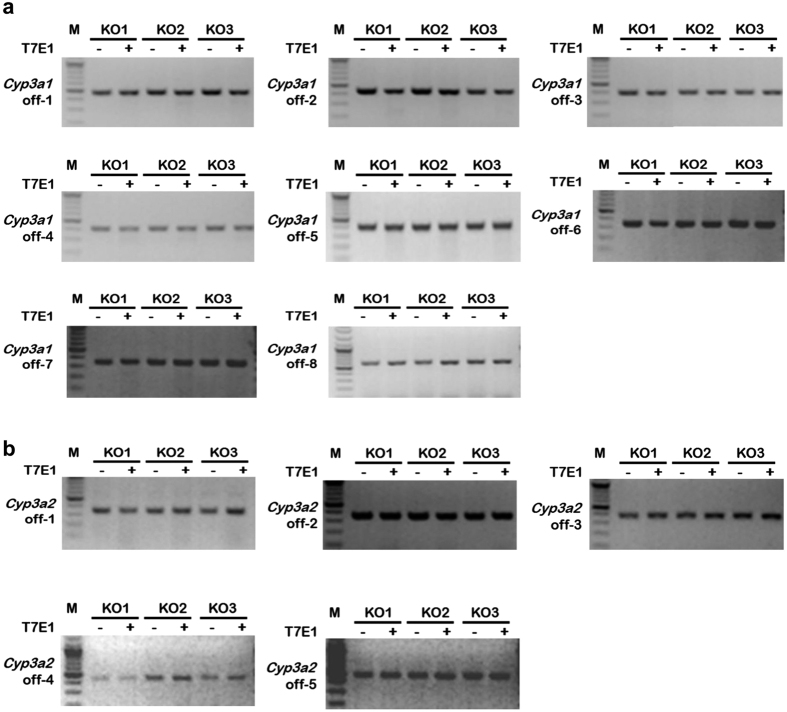
Off-target analysis of CRISPR-Cas9 induced mutation in three (**a**) *Cyp3a1* and (**b**) *Cyp3a2* double KO rats. Eight OTSs for *Cyp3a1 s*gRNA and five OTSs for *Cyp3a2* sgRNA were selected for T7E I enzyme cleavage. No mutations were found in PCR products, suggesting that CRISPR-Cas9 did not disrupt the off-target sequences tested in this study. *Cyp3a1* off, OTS for *Cyp3a1* sgRNA; KO, *Cyp3a1* and *Cyp3a2* double KO rat; T7E I−, before T7E I digestion; T7E I+, after T7E I digestion; M, DNA molecular weight marker.

Recent studies have reported that the CRISPR-Cas9 system has a much greater level of off-target cleavage than zinc-finger nucleases (ZFNs) and transcription activator-like effector nucleases (TALENs) because the target sequence selected by CRISPR-Cas9 can tolerate a 1~3 base pair mismatch^8^,^10^,^15^,^16^,^17^. Hence, we examined off-target damage in *Cyp3a1* and *Cyp3a2* double KO rats. A genome-wide sgRNA off-target searching tool (COD) was used to pick out off-target sites (OTS) with high potential for mutation by our targeting system. OTS with a score of no less than 0.5 was chosen for further analysis. We examined eight and five OTSs for *Cyp3a1* and *Cyp3a2* sgRNAs, respectively (Table 1). None of these potential OTSs was mutated in our double KO rats (Fig. 3), thus suggesting CRISPR-Cas9 is a reliable gene targeting tool in generating the *Cyp3a1/2* double KO rat model.

### Physiological phenotype of *Cyp3a1* and *Cyp3a2* double KO rat and WT rat

**Figure 4:**
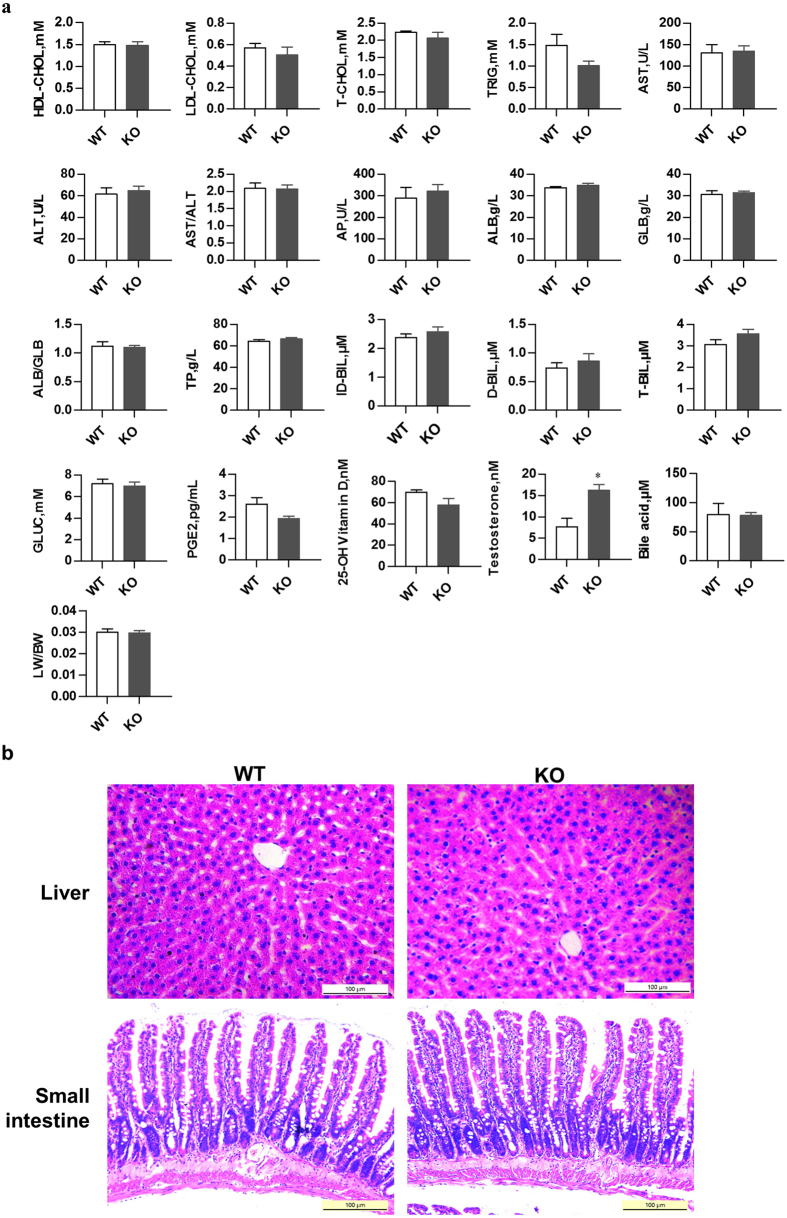
Physiological phenotype of *Cyp3a1/2* double KO rats. (**a**) Clinical chemistry and physiological analysis of *Cyp3a1/2* double KO rat serum compared with WT rats. Serum samples were collected at nearly 8 weeks and analyzed for HDL-CHOL, LDL-CHOL, T-CHOL, TRIG, AST, ALT, AST/ALT, AP, ALB, GLB, ALB/GLB, TP, ID-BIL, D-BIL, T-BIL, GLUC, PEG2, 25-OH vitamin D, testosterone and bile acid. Organ coefficient of *Cyp3a1/2* double KO rat (about 8 weeks) liver compared with WT rats. LW, liver weight; BW, body weight. The organ coefficient was defined as LW/BW ratio. Data were shown as Mean ± SEM of six rats. (**b**) HE staining of liver and small intestine slices from WT and *Cyp3a1/2* double KO rats (n = 6). Scale bar = 100 μm.

CYP3A1 and CYP3A2 enzymes were involved in the metabolism of xenobiotics, steroid bile acids and other compounds. Therefore, the deletion of *Cyp3a1* and *Cyp3a2* may lead to important physiological changes. To investigate the effects of *Cyp3a1* and *Cyp3a2* disruption on rat physiology, serum samples were collected at 8 weeks and analyzed for high-density lipoproteins-cholesterol (HDL-CHOL), low-density lipoproteins-cholesterol (LDL-CHOL), total cholesterol (T-CHOL), triglycerides (TRIG), aspartate amino transferase (AST), alanine amino transferase (ALT), AST/ALT, alkaline phosphatase (AP), albumin (ALB), globulin (GLB), ALB/GLB, total proteins (TP), ID-BIL (indirect bilirubin), direct bilirubin (D-BIL), total bilirubin (T-BIL), glucose (GLUC), prostaglandin E2 (PGE2), 25-OH vitamin D, testosterone and bile acid. None of the serum clinical chemistry and physiological indices showed obvious abnormalities except for testosterone (Fig. 4a). The concentration of testosterone (a typical substrate of CYP3A) increased by 110% in the serum of KO rats. Compared with WT, homozygous *Cyp3a1* and *Cyp3a2* double KO rats seemed to be normal and fertile, with regular liver weight, body weight as well as organ coefficients for both genders (Fig. 4a). Furthermore, histological analysis revealed that there were no morphological changes between the WT and KO rat liver and small intestine (Fig. 4b). In general, the absence of *Cyp3a1* and *Cyp3a2* may not cause any consequential abnormalities.

### Double KO rats lack expression of CYP3A1 and CYP3A2

**Figure 5:**
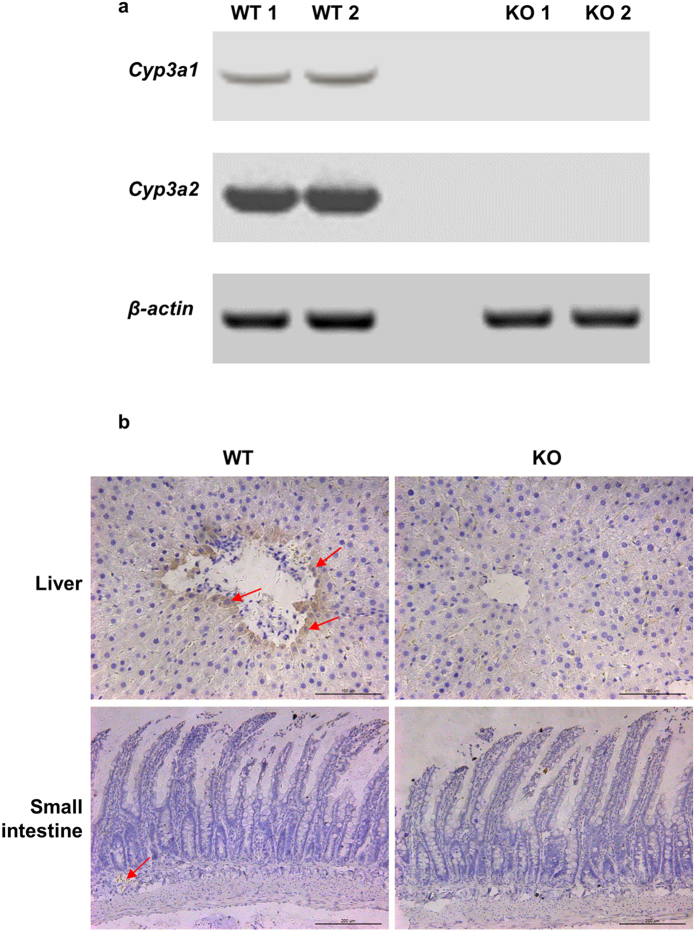
mRNA and immunohistochemical detection of *Cyp3a1* and *Cyp3a2* in double KO and WT male rat liver and small intestine. (**a**) Hepatic mRNA of WT rats and KO rats was detected by using agarose gel electrophoresis. *β-actin* was set as the reference gene. (**b**) Liver and small intestine sections were used for immunohistochemical staining. Experiments were repeated three times independently.

The expression of CYP3A1 and CYP3A2 in mRNA levels in double KO and WT rat liver was checked via the specific primer pairs targeting *Cyp3a1* and *Cyp3a2*. Our data showed that the mRNA expression of *Cyp3a1* and *Cyp3a2* was completely absent in double KO rat livers (Fig. 5a). We also found no CYP3A1 or 3A2 expression by immunohistology in liver and small intestine of double KO rats (Fig. 5b). These results confirmed that the expression of CYP3A1 and CYP3A2 was lost in the double KO rat liver and intestine.

### CYP3A-mediated metabolism and pharmacokinetics studies in Cyp3a1/2 double KO rats

**Figure 6:**
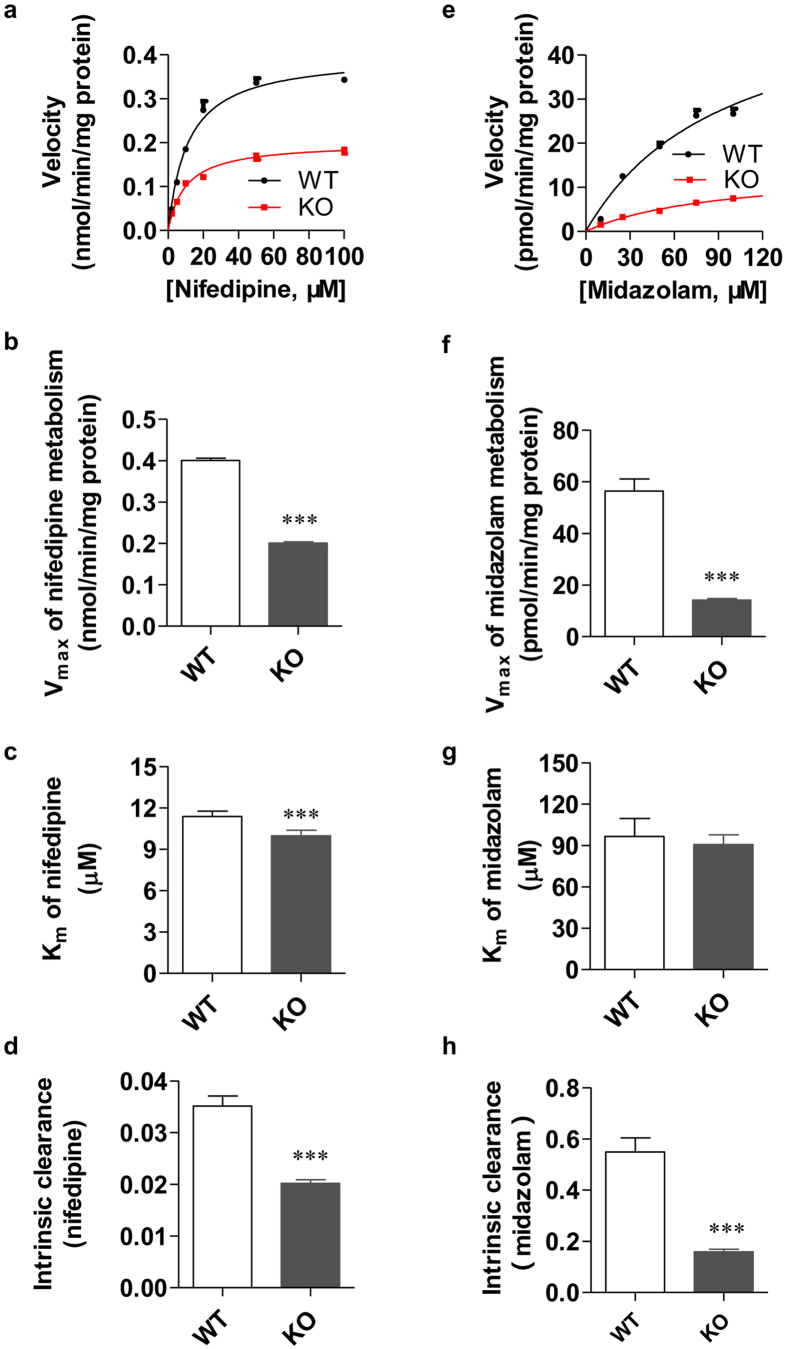
CYP3A-mediated metabolic activity in *Cyp3a1/2* double KO and WT rats *in vitro*. (**a**) The saturation curve of nifedipine metabolism in liver microsomes from both double KO and WT rats. (**b**) The V_max_ values of nifedipine metabolism in both double KO and WT male RLM. (**c**) The K_m_ values of nifedipine metabolism in both double KO and WT male RLM. (**d**) The CL_int_ values of nifedipine metabolism in both double KO and WT male RLM. (**e**) The saturation curve of midazolam metabolism in liver microsomes from both double KO and WT male rats. (**f**) The V_max_ values of midazolam metabolism in both double KO and WT male RLM. (**g**) The K_m_ values of midazolam metabolism in both double KO and WT male RLM. (**h**) The CL_int_ values of midazolam metabolism in both double KO and WT male RLM. All data were expressed as mean ± SEM of six rats in each group, and ***p < 0.001 compared to WT rats.

To assess whether CYP3A1/2 was functionally inactive in KO rats, the *in vitro* (substrate: midazolam and nifedipine) and *in vivo* (substrate: nifedipine) metabolic studies of CYP3A1/2 substrates were carried out. In *in vitro* studies, the maximum velocity (V_max_) of dehydronifedipine formation in rat liver microsomes (RLM) of double KO rats was 0.20 ± 0.01 nmol/min/mg protein, a significant decrease (about 50%) of that in WT controls (0.40 ± 0.01 nmol/min/mg protein) (Fig. 6a,b). The Michaelis constant (K_m_) value of KO RLM was 9.97 ± 1.13 μM, which was slightly decreased by 12% compared with that in WT RLM (11.37 ± 1.10 μM) (Fig. 6c). Meanwhile, the intrinsic clearance (CL_int_) of nifedipine in the RLM of double KO rats significantly decreased by 43% compared with that in WT RLM (Fig. 6d). Moreover, the V_max_ and CL_int_ of midazolam in the RLM of double KO rats also significantly decreased by 75% and 70%, respectively, compared with those in WT RLM (Fig. 6f,h). In summary, the CYP3A1/2 activity of KO rats was significantly decreased in our metabolic studies of the CYP3A substrates nifedipine and midazolam *in vitro*.

To further explore the impaired function of CYP3A1/2 in *Cyp3a1/2* double KO rats *in vivo*, a single dose of nifedipine (200 μg/kg) was administered through the tail vein in both KO and WT rats. As shown in Table 2, the area under the time-plasma concentration curve (AUC) of nifedipine in double KO rats was increased by 61% compared with that in WT rats. Furthermore, in KO rats the elimination half-time (t_1/2_) was significantly prolonged by 93%, together with a 25% increase in the mean residence time (MRT) and a 50% decrease in the clearance (CL), compared with these in WT rats. These data reflected that the function of CYP3A1/2 in KO rats was impaired *in vivo*. Therefore, the pharmacokinetic results of nifedipine *in vivo* were in agreement with the *in vitro* results, indicating that CYP3A1/2 was functionally inactive in the double KO rat line.

### Compensatory expression of other CYP isoforms in *Cyp3a1/2* KO rats

**Figure 7:**
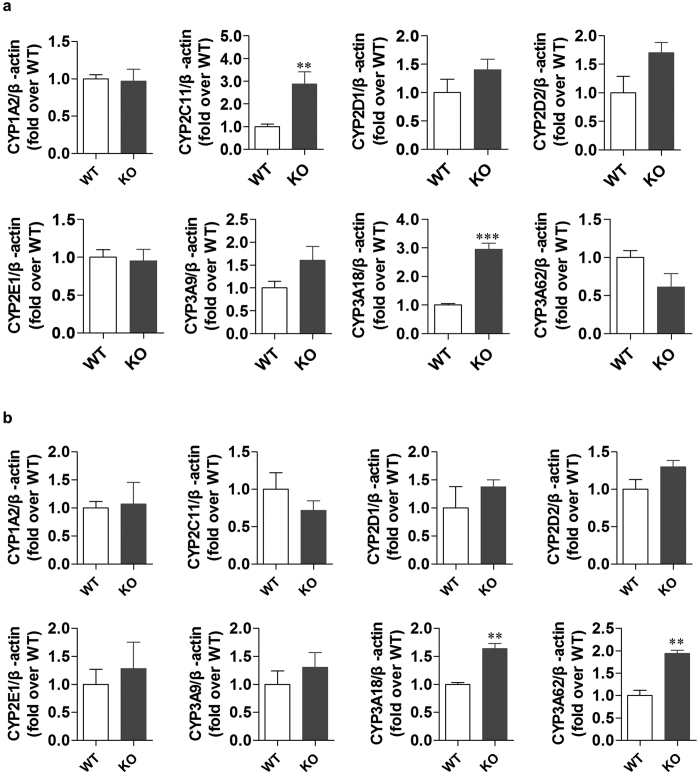
The mRNA expression levels of the other main CYP isoforms in *Cyp3a1/2* double KO and WT rats. The expression of other isoforms was measured in rat liver (**a**) and small intestine (**b**). *β-actin* was used as the reference gene and was stably expressed in both WT and KO rat liver and small intestine. All data were expressed as mean ± SEM (n = 5), and **p < 0.05, ***p < 0.001 compared to WT controls.

Since *Cyp3a1/2* deletion may affect the expression of other CYP isoforms, the mRNA levels of the other main rat CYP enzymes were checked via quantitative real-time PCR. Compared with WT rats, no significant change was observed in the hepatic and small intestinal expression of CYP1A2, CYP2D1, CYP2D2, CYP2E1, and CYP3A9 (Fig. 7). In contrast, the hepatic mRNA expression of CYP2C11 and CYP3A18 in double KO rats was increased by nearly 290% and 300%, respectively, compared with WT controls (Fig. 7). Meanwhile, in small intestine of KO rats, the expression of CYP3A18 and CYP3A62 was up-regulated by 60% and 93%, respectively (Fig. 7).

## Discussion

CYP3A as the most abundant human liver CYP enzyme is not only involved in the biotransformation of many endogenous substances, such as fatty acids, eicosanoid sterols, bile acids and vitamin D^4^, but also participates in the metabolism of over 75% of clinical drugs^18^. Traditionally, the roles of CYP3A in metabolism and metabolism-based toxicity have been investigated using specific antibodies or ‘selective’ chemical inhibitors, but in later studies most of these ‘specific’ modulators turned out to be nonspecific and even toxic under physiological conditions^19^,^20^. Therefore, a novel *Cyp3a* KO animal model is needed to study the functions of CYP3A *in vivo*. To our knowledge, the present work is the first time to successfully create a *Cyp3a1/2* double KO rat model using CRISPR-Cas9 system.

Traditional gene targeting via embryonic stem cells to generate specific modified alleles is a potent tool to illustrate functions of genes in mice^21^. In rats, however, this genome manipulation method in stem cells does not work because of some technical difficulties^10^,^22^. In recent years, the emergences of engineered nucleases, such as ZFNs and TALENs, have been used for gene editing in rats. However, they are still limited by the time- and labor-consuming construction of engineered specific protein pairs for every target site^10^. More recently, a state-of-the-art engineered nuclease, the CRISPR-Cas9 system, has proved a simple method to manipulate genes in rats^8^,^10^,^13^. In particular, we have used the CRISPR-Cas9 tool to successfully knock out the *Cyp2e1* gene from the rat^12^.

In rats, the high degree of nucleotide similarity between CYP3A1 and 3A2 presents a major challenge towards simultaneous disruption of these isozymes. However, compared with previous techniques such as ZFNs and TALENs, the CRISPR-Cas9 system displays distinct advantages in editing multiple genes simultaneously^10^,^13^. Therefore, we targeted the *Cyp3a1* and *Cyp3a2* genes simultaneously via the CRISPR-Cas9 system. In this study, the CRISPR-Cas9 tool showed great advantages again in successfully achieving precision gene targeting in the rat by co-microinjection of *Cyp3a1* and *Cyp3a2* sgRNA with Cas9 mRNA into zygotes.

The *Cyp3a1/2* double KO rats were viable and fertile. At first, we thought simultaneous KO *Cyp3a1/2* might show disruptive effects on rat physiology. However, our data indicated that *Cyp3a1/2* double KO rats possessed equivalent levels of cholesterol, aminotransferase, glucose, bilirubin, PEG2, 25-hydroxy vitamin D, bile acid as well as serum proteins (Fig. 4). These results revealed that CYP3A may work as an important rather than exclusive contributor to the metabolism of endogenous substances, which is also in agreement with its role in the *Cyp3a* KO mouse model^3^. Recently, it has been reported that *Cyp3a* KO mice showed higher levels for bile acid and testosterone compared with WT mice^23^,^24^. In contrast, *Cyp3a* KO rats only presented higher level of testosterone than that of WT rats, thus suggesting different species possess different metabolic characterization. Moreover, some studies have reported that the deletion of one *Cyp* gene in mouse may lead to compensatory changes in other CYP metabolic enzymes^25^. Therefore, the mRNA levels of other main CYP isoforms were quantified in this study. Our data exhibited there were no statistically significant changes in most CYP enzymes, apart from a compensatory increase of *Cyp2c11* (2.9-fold) and *Cyp3a18* (3-fold) mRNA in liver, *Cyp3a18* (1.63-fold) and *Cyp3a62* (1.93-fold) in small intestine (Fig. 7). The up-regulation of *Cyp2c11, Cyp3a18* and *Cyp3a62* may be owed to the ligand-activated transcription factor, which is known as the pregnane X receptor (PXR) or pregnane-activated receptor^26^,^27^. Our further study showed that the mRNA level of PXR was about 1.5-fold higher in KO rat liver compared with WT rats (data not shown). Due to the up-regulation of *Cyp3a18* and *3a62* in KO rats, it would be better to delete all of CYP3A isoforms in the rat to completely eliminate the *Cyp3a* background^28^. In spite of these limitations, the *Cyp3a1/2* KO rat model is still a novel and useful animal model to explore the physiological and pharmacological roles of CYP3A and to assess whether the absence of CYP3A could arouse compensatory metabolism by non-CYP enzymes or other CYP isoforms in rats. For example, in this study, there was discrepancy of midazolam between *in vitro* metabolic study using liver microsomes and *in vivo* pharmacokinetic study in WT rats. Therefore, it is difficult to decide whether midazolam has the compensatory metabolic pathways *in vitro* and *in vivo*. However, a CYP2C specific inhibitor or antibody may be effective to explore the compensatory effect of CYP2C in the metabolism of midazolam when *Cyp3a1/2* KO rat model is used.

The *Cyp3a1/2* double KO rat model can be used *in vivo* to study CYP3A-mediated drug metabolism and pharmacokinetics. In this study, nifedipine and midazolam as CYP3A substrates were used to detect the CYP3A-mediated catalytic activity in WT and double KO rats. RLM from double KO rats showed a decreased *in vitro* metabolic activity both on nifedipine and midazolam (Fig. 6), suggesting the loss of CYP3A. However, residual metabolites of nifedipine and midazolam were still formed by RLM from double KO rats, which may be ascribed to other hepatic CYP isoforms and/or the compensatory up-regulation of other CYP isoforms. For example, previous studies have reported that midazolam is the probe substrate of CYP3A, but CYP2C also takes part in its metabolism^25^,^29^,^30^. In this study, we compared the pharmacokinetic profiles of midazolam between CYP3A1/2 KO and WT rats. However, there was no obvious difference of the pharmacokinetics of midazolam between KO and WT rats. Therefore, we used nifedipine instead of midazolam in the *in vivo* pharmacokinetic studies. The results *in vivo* showed that the exposure of nifedipine (AUC value) in *Cyp3a1/2* KO rats significantly increased by 61%, together with increases in the t_1/2_ (93%) and MRT (25%), compared with WT rats (Table 2). Our findings demonstrated that *Cyp3a1/2* double KO rats were a sensitive animal model to investigate the CYP3A-mediated metabolic pathway of chemicals, especially in *in vivo* studies. Given that a discrepancy in CYP3A exists between rats and humans, a CYP3A humanized rat model should be further created to overcome the species differences.

In conclusion, the CRISPR-Cas9 method was described to successfully create the *Cyp3a1/2* double KO rat model. The *Cyp3a1*/2 double KO rats were viable, fertile, and physiological normal. The *Cyp3a1/2* double KO rats are a valuable animal model to investigate the roles of CYP3A in drug and chemical metabolism, toxicity and carcinogenicity *in vivo*.

## Methods

### Materials and chemicals

Oligos (60 bp, containing *Cyp3a1* or *Cyp3a2* knock out target-sites) and all primers for PCR were synthesized from Biosune Biotechnology Co. LTD (Shanghai, China). *In vitro* Transcription T7 Kit, SYBR Premix Ex Taq, Prime Script RT Reagent Kit and TA cloning kit were bought from Takara (Dalian, China). mMessage mMachine SP6 kit were purchased from Thermo Scientific (Massachusetts, USA). The secondary antibody conjugated to HRP-labeled polymers was bought from Mrbiotech (Shanghai, China). Midazolam was purchased from Enhua (Nanchang, China). Mebendazole was purchased from Aladdin (Shanghai, China).

### Animals

Male and female SD rats (8-week old) were purchased from National Rodent Laboratory Animal Resources, Shanghai Branch of China. The animals were kept in a specific pathogen-free facility with access to rodent chow cubes and sterile water, with 12 h light-dark cycles. All the methods performed in animals were carried out in accordance with the National Institutes of Health standards established in the ‘Guidelines for the Care and Use of Experimental Animals’. All experimental protocols in animals were approved by the Ethics Committee on Animal Experimentation of East China Normal University (Shanghai, China).

### *Cyp3a1* and *Cyp3a2* KO target site selection

The sequence fragments of *Cyp3a1* and *Cyp3a2* were submitted to an online design tool (CRISPR Design Tool, http://tools.genome-engineering.org) to generate the potential target sites. *Cyp3a1* and *Cyp3a2* in rats contain 13 and 10 exons, respectively. To silence the *Cyp3a1* and *Cyp3a2* gene as completely as possible, target-sites were selected just downstream to the initiation codon, ATG, and followed by a protospacer adjacent motif (PAM) site (5′-NGG-3′) in the 3′ end.

### DNA constructs and *in vitro* transcription

The sgRNA expression vector and Cas9-encoding plasmid were constructed according to our previous work^14^. Then oligos (60 bp, containing *Cyp3a1* or *Cyp3a2* knock out target-sites) were cloned into the pGS3-T7-gRNA vector through overlapping PCR for the transcription of sgRNA *in vitro. Cyp3a1* and *Cyp3a2* sgRNA were then transcribed with the *in vitro* T7 Transcription Kit respectively. RNA was purified through phenol/chloroform extraction and dissolved in diethylpyrocarbonate-treated or RNase-free water.

For the transcription of Cas9 mRNA, the linearized Cas9 expression vectors were purified through phenol/chloroform extraction and ethanol precipitation followed by the transcription of Cas9 mRNA using the mMessage mMachine SP6 kit. The mRNA was recovered by Lithium chloride precipitation and resuspended in Nuclease-free water. Purified *Cyp3a1* and *Cyp3a2* sgRNA and Cas9 mRNA products were confirmed by electrophoresis.

### Co-microinjection of sgRNA and Cas9 mRNA into zygotes

Rat preparation and microinjection were performed as described with modifications^14^. Briefly, the TE buffer containing 25 ng/μL of *Cyp3a1* sgRNA, 25 ng/μL of *Cyp3a2* sgRNA and 50 ng/μL of Cas9 mRNA were co-injected into the cytoplasm of one-cell stage embryos. The microinjected zygotes were transferred into pseudopregnant female rats immediately after injection or after overnight culture in embryo culture medium.

### Genotyping of founders and progenies

For the F_0_ generation, newborn rats were genotyped 7 to 10 days after birth. Purified genomic DNA was amplified using the primers listed in Table 3 (No. 1, 2). T7E I assay was used for a preliminary screening for the potential mutations in founders on target-sites. To identify the modification details in founders, containing potential mutations, PCR products from each founder was cloned into pMD-18T vectors for sequencing with the universal primers of the vector. For the F_1_ and F_2_ generations, the genotype was identified by sequencing the PCR products directly. Sequence data was analyzed through DNAMAN (LynnonBiosoft, CA, USA) to identify the exact genotype (WT, heterozygote and double KO rats) of founders. Specificity of all primer pairs (No. 1 to No. 26) used in our research was checked using agarose gel electrophoresis, indicating a single and specific band for each pair of primer under our experimental conditions.

### Off-target site validation

The target sites of *Cyp3a1* and *Cyp3a2* were submitted to Cas9 online designer (http://cas9.wicp.net/). The degree of off-target was measured by an ‘off-target score’, which ranges from 1 to 0. ‘1’ means a high probability of off target and ‘0’ a low probability of off target events. OTS with a score ≥0.5 were selected for PCR analysis. The PCR product of each off-target site was subjected to T7E I digestion and then resolved on a 1.5% agarose gel to analyze the off-target effects.

### Hematoxylin and eosin (HE) staining of rat liver and small intestine slices

About 8-week old rats were sacrificed through cervical dislocation. Livers and intestines were freshly excised and then fixed in 4% paraformaldehyde at 4 °C for more than 12 h. The fixed intestines were dehydrated in 50%, 75%, 85%, 95%, and 100% ethyl alcohol for one hour for each and then immersed in ethyl alcohol and xylene mixture (v/v = 1:1) for 30 min, followed by two 15 min intervals in xylene, followed by paraffin embedding. The fixed livers similarly treated, except dehydration for 30 min per step and 15 minute immersion in the ethanol:xylene mixture. Sections (4 μm) were then deparaffinization for hematoxylin and eosin (H&E) according to normal procedures.

### Clinical-chemical and hematological analysis of *Cyp3a1* and *Cyp3a2* double KO and WT rats

To further characterize the potential effects of *Cyp3a1* and *Cyp3a2* KO on the normal rat physiology, the serum samples were collected for clinical-chemical and hematological analysis. The serum samples were sent for analysis by ADICON Clinical Laboratories (Shanghai, China). For the analysis of PGE2, a rat PEG2 ELISA kit was bought from Shanghai MLBIO Biotechnology Co.Ltd (Shanghai, China).

### *Cyp3a1* and *Cyp3a2* mRNA expression in rat liver

About 8-week old rats were sacrificed through cervical dislocation method. Livers mRNA was extracted through Trizol and the RNA was reverse-transcribed into cDNA using the Takara RR036A RT kit. For the detection of *cyp3a1* and *cyp3a2* mRNA expression, selective primers (spanning different exons) were designed and synthesized which were listed in Table 3 (No. 24, 25). *β-actin* (Primer listed in Table 3, No. 26) was used as the internal reference.

### Immunohistochemical analysis

Immunohistochemistry on double KO and WT rat liver and small intestine was conducted with a commercial rabbit anti-human CYP3A4 polyclonal antibody (1:100, ab3572, Abcam), which cross-reacts with rat CYP3A, and a secondary antibody conjugated to HRP-labeled polymers (MR-SPR120, Mrbiotech).

### Quantitative reverse transcriptase PCR

SYBR-PCR was performed using a Stratagene Mx3005P with SYBR Premix Ex Taq. The relative mRNA expression was measured by 2^−ΔΔCt ^31. The primers (spanning different exons) used for quantitative reverse transcription-PCR were listed in Table 3 (No. 16–23, 26). Dissociation curves of primer pairs for quantitative reverse transcriptase-PCR were monitored, showing a high specificity for our primer pairs.

### Preparation of RLMs

The protocol of RLM preparation was modified from our previous studies^32^,^33^. The liver was separated, rinsed with ice-cold normal saline and homogenized in a 0.05 M Tris/KCl buffer (pH 7.4). The homogenate was centrifuged at 10,500 g at 4 °C for 30 min. The supernatant was then centrifuged at 105,000 g at 4 °C for 60 min. The precipitate was resuspended and re-centrifuged at 105,000 g at 4 °C. The pellet was reconstituted with the 0.05 M Tris/KCl buffer (pH 7.4) and stored in −80 °C for use.

### The CL_int_ of nifedipine and midazolam in *Cyp3a1* and *Cyp3a2* double KO and WT RLM

For the research of CYP3A1 and CYP3A2 enzymes activity *in vitro*, nifedipine and midazolam were chosen as specific substrates and dehydronifedipine and 1′-hydroxymidazolam were monitored, respectively^34^,^35^,^36^,^37^. The incubation mixture consisted of an NADPH (β-Nicotinamide adenine dinucleotide phosphate hydrate)-regenerating system, nifedipine (2 μM to 100 μM) or midazolam (10 μM to 100 μM) and 0.5 mg/mL (for nifedipine) or 1 mg/mL (for midazolam) of RLM in 0.05 M Tris-HCl buffer (pH 7.4). The supernatant of the incubation mixture was transferred to the autosampler vial after a protein precipitation process for LC-MS/MS analysis. K_m_ and V_max_ were analyzed according to the standard Michaelis-Menten equation by GraphPad Prism 5. The CL_int_ was defined as the V_max_/K_m_ ratio.

### Pharmacokinetics of nifedipine in *Cyp3a1* and *Cyp3a2* double KO and WT rats

Nifedipine (200 μg/kg) was administered through the tail vein for all WT and KO rats and blood samples were collected into heparinized centrifuge tubes at 2, 5, 10, 20, 30, 45, 60, 90, and 120 min by orbital bleeding with capillary tubes. Blood samples were centrifuged as soon as possible at 5,500 g for 15 min, and the plasma was transferred into new tubes and frozen at 0 °C for further analysis.

### LC-MS/MS analysis of 1’-hydroxymidazolam, nifedipine and dehydronifedipine

For the LC-MS/MS analysis, a 1290 HPLC-6460 triple quadrapole mass spectrometer coupled with an ESI ion source (Agilent Technologies, USA) was employed. For 1′-hydroxymidazolam detection, a Zorbax Eclipse C18 column (2.1*50 mm, 1.8 μm; Agilent Technologies, USA) was used with a mobile phase system of water (A)-acetonitrile (B). The ion transitions of 342.0 → 324.0 and 285.0 → 193.1 for 1′-hydroxymidazolam and diazepam (IS) were monitored in positive ESI mode, respectively.

For the detection of nifedipine in rat plasma, the mobile phase consisted of water (A) and acetonitrile (B). The detection of the ions was performed in the multiple reactions monitoring (MRM) mode, monitoring the transition of m/z 347.1 to the m/z 315.1 product ion for nifedipine, and m/z 296.1 precursor to m/z 264.1 product ion for mebendazole (IS). The liquid–liquid extraction method was used to isolate nifendipine and IS from plasma.

For the detection of dehydronifedipine, a Phenomenex Kinetex XB-C18 column (100 × 3.00 mm, 2.6 μm) was used. The gradient elution was conducted with a mobile phase system of water (A)-acetonitrile (B). The ion transitions of 345.3.0 to 284.1 and 296.1.0 to 264.1 for dehydronifedipine and mebendazole (IS) were monitored, respectively.

### Data analysis and statistics

All data were presented as mean ± SEM. Statistical analysis between different groups was performed using two-tailed t-test and *p*-values less than 0.05 were considered to indicate statistical significance. The enzyme kinetic data of nifedipine and midazolam metabolism in RLM was fitted according to the typical Michaelis-Menten equation with GraphPad Prism 5.0 (GraphPad Software, CA, USA). Pharmacokinetic parameters were calculated by WinNonlin software version 5.2.1 (Pharsight Corporation, Mountain View, USA) based on non-compartmental analysis.

## Additional Information

**How to cite this article**: Lu, J. *et al*. CRISPR knockout rat cytochrome P450 3A1/2 model for advancing drug metabolism and pharmacokinetics research. *Sci. Rep.* **7**, 42922; doi: 10.1038/srep42922 (2017).

**Publisher's note:** Springer Nature remains neutral with regard to jurisdictional claims in published maps and institutional affiliations.

## Figures and Tables

**Table 1:** Details for potential off-target sites examined.

Match Name	Coordinate	Spacer + PAM	Number of mismatch	off-target score
*Cyp3a1* sgRNA		CAAGAAACAGGGGATTCCTGG		
*Cyp3a1*-off-1	Chr12: 12839905 to 12839886	AAGAAACAGGGaATTCCTGG	1	0.70
*Cyp3a1*-off-2	Chr12: 13740208 to 13740189	AAGAAACAaGGaATTCCTGG	2	0.56
*Cyp3a1*-off-3	Chr12: 17502003 to 17501985	AGAgACAGGGGATcCCTGG	2	0.50
*Cyp3a1*-off-4	Chr12: 43976656 to 43976638	AGAgACAGGGGATcCCaGG	3	0.50
*Cyp3a1*-off-5	Chr12: 16851108 to 16851124	AAACAGGGGATcCCgGG	2	0.50
*Cyp3a1*-off-6	Chr12: 41792250 to 41792265	AACAGGGGATTCCaGG	1	0.50
*Cyp3a1*-off-7	Chr12: 5140566 to 5140580	ACAGGGGATTCCaGG	1	0.50
*Cyp3a1*-off-8	Chr13: 50280364 to 50280382	AGAcACAGGGGATTCCTGG	1	0.50
*Cyp3a2* sgRNA		TAAGAAACAAGGAATTCCTGG		
*Cyp3a2*-off-1	Chr12: 12747958 to 12747978	TAAGAAACtAGGAATTCCTGG	1	0.70
*Cyp3a2*-off-2	Chr12: 12839906 to 12839886	TAAGAAACAgGGAATTCCTGG	1	0.70
*Cyp3a2*-off-3	Chr12: 13172446 to 13172427	AAGAAACAgGGgATTCCTGG	2	0.63
*Cyp3a2*-off-4	Chr12: 52934974 to 52934956	AGAtACcAGGAATTCCaGG	3	0.56
*Cyp3a2*-off-5	Chr12: 40658157 to 40658141	AAAgAAGGAATTCCTGG	1	0.50

**Table 2:** Pharmacokinetic parameters of nifedipine in double KO and WT rats.

Pharmacokinetic parameter	WT	KO
t_1/2_ (min)	35.52 ± 8.29	68.42 ± 9.23***
C_0_ (ng/mL)	926.52 ± 142.87	870.74 ± 204.87
AUC_0–120 min_ (min·ng/mL)	26979.23 ± 1522.03	43338.98 ± 7259.09**
AUC_0−∞_ (min*ng/mL)	30118.55 ± 2436.55	61124.74 ± 10417.95***
V_d_ (mL/kg)	341.11 ± 83.37	332.07 ± 80.81
CL (mL/min/kg)	6.67 ± 0.51	3.36 ± 0.68***
MRT (min)	38.06 ± 5.41	47.74 ± 2.21**

t_1/2_, half life; C_0_, initial plasma concentration; AUC_0–120min_, area under the plasma concentration-time curve during 0–120 min; AUC_0–∞_, area under the plasma concentration-time curve during 0−∞; V_d_, apparent volume of distribution; CL, clearance; MRT, mean residence time. All data were expressed as mean ± SEM (n = 5). **p < 0.01 and ***p < 0.001 compared with WT rats.

**Table 3:** Primer pairs used in the research.

No.	Primer Name	Primer Sequence (5′ → 3′)	No.	Primer Name	Primer Sequence (5′ → 3′)
1	*Cyp3a1*-genotyping-S	TAGCATTACCCTGGCACCT	14	*Cyp3a2*-off-4-S	GCGGGTGGAAGAAGAGTG
	*Cyp3a1*-genotyping-A	GCCAAAGCCTGGATACACTC		*Cyp3a2*-off-4-A	TTGTGCAGAGCCAGATGC
2	*Cyp3a2*-genotyping-S	TAGAGGGAGAACACCGAGGAG	15	*Cyp3a2*-off-5-S	TATCAACTACTGTTTCCCAATG
	*Cyp3a2*-genotyping-A	GGGTCCGATGTCTTAGGGTT		*Cyp3a2*-off-5-A	TGTAACTTGCGTGTAGACTTT
3	*Cyp3a1*-off-1-S	AGACCCAAGGGACTCAAA	16	*Cyp1a2*-qPCR-S	ATGTCACCTCAGGGAATG
	*Cyp3a1*-off-1-A	AACATCCACCAGCAGTTT		*Cyp1a2*-qPCR-A	CACCGTTGTCTTTGTAGTTC
4	*Cyp3a1*-off-2-S	CCAGGGACTCACAAACAT	17	*Cyp2c11*-qPCR-S	AAAGCACAATCCGCAGTC
	*Cyp3a1*-off-2-A	GAACCCAAACATCCACAT		*Cyp2c11*-qPCR-A	CATCCGTGTAGGGCATCT
5	*Cyp3a1*-off-3-S	TGGAAGCCAGAGGTTGAT	18	*Cyp2d1*-qPCR-S	ATGATTCTATACCCGGATGTG
	*Cyp3a1*-off-3-A	TTCCCACAGTCTGCCATT		*Cyp2d1*-qPCR-A	ACGGACGACAGGTTGATG
6	*Cyp3a1*-off-4-S	TGTGTCTATGTTGTGTATATCTGGG	19	*Cyp2d2*-qPCR-S	GCAGGTGGACTTTGAGAAC
	*Cyp3a1*-off-4-A	CGGCAGGAAGAGATAAATGACAGAT		*Cyp2d2*-qPCR-A	GATTATAGATGGGCAGTAGGG
7	*Cyp3a1*-off-5-S	GTGAGCTGCCCATGTTAA	20	*Cyp2e1*-qPCR-S	GATCTATAACAGTTGGAACCTGCCCC
	*Cyp3a1*-off-5-A	ATCCCATCTGATCTGTCG		*Cyp2e1*-qPCR-A	CAGGACCACGATGCGCCTTGAGCCA
8	*Cyp3a1*-off-6-S	ACACTGCCCTAACGCTCAT	21	*Cyp3a9*-qPCR-S	CTGTGGGTTGTTAAGGGAA
	*Cyp3a1*-off-6-A	CACGCCCTCACCTAATCT		*Cyp3a9*-qPCR-A	TGAGGCAGGGATCGGAGGA
9	*Cyp3a1*-off-7-S	GAGGGCAGTTTCAGGTAT	22	*Cyp3a18*-qPCR-S	ACCCAAACCTGTGCCTTTA
	*Cyp3a1*-off-7-A	TCTTTGGAGGGTCTTTCT		*Cyp3a18*-qPCR-A	CACATGCCATCACCGTAG
10	*Cyp3a1*-off-8-S	CAATGGGATTTGTGGAGG	23	*Cyp3a62*-qPCR-S	TGCTCCTGTATCTGTATGG
	*Cyp3a1*-off-8-A	CACCCTTTTCTCACAGCTCCT		*Cyp3a62*-qPCR-A	ATGGCAGTGTCTATCAAAC
11	*Cyp3a2*-off-1-S	CGTCGGGTTTGATTCTTG	24	*Cyp3a1*-cDNA-S	GCTGAATTACTATATGGGTT
	*Cyp3a2*-off-1-A	CAGCACTTGGGAGACAGA		*Cyp3a1*-cDNA-A	TCATGATCCAGTTATGATTT
12	*Cyp3a2*-off-2-S	AGACCCAAGGGACTCAAA	25	*Cyp3a2*-cDNA-S	TAGGCACTGTGCTGAATTAC
	*Cyp3a2*-off-2-A	GGAGGAGCAGCCAATCGT		*Cyp3a2*-cDNA-A	TCAGGCTCCATTTATGAC
13	*Cyp3a2*-off-3-S	TGATGTCTCAGGGTTTCC	26	*β-actin*-S	GTACGCCAACACAGTGCTG
	*Cyp3a2*-off-3-A	TCTAGTTATCCCTAGTTCCA		*β-actin*-A	CGTCATACTCCTGCTTGCTG
